# Work ability assessment in a worker population: comparison and determinants of Work Ability Index and Work Ability score

**DOI:** 10.1186/1471-2458-13-305

**Published:** 2013-04-08

**Authors:** Mehdi El Fassi, Valery Bocquet, Nicole Majery, Marie Lise Lair, Sophie Couffignal, Philippe Mairiaux

**Affiliations:** 1 Public Health Department, Liège University, Liège, Belgium; 2 Service de Santé au Travail Multisectoriel (STM), Strassen, GD, Luxembourg; 3 Centre for Health Studies (CRP Santé), Strassen, GD, Luxembourg; 4 Centre for Health Studies (CRP Santé), Competences Centre for Methodology and Statistics, Strassen, GD, Luxembourg

**Keywords:** Ageing workers, Health surveillance, Occupational health, Perceived health, Work ability

## Abstract

**Background:**

Public authorities in European countries are paying increasing attention to the promotion of work ability throughout working life and the best method to monitor work ability in populations of workers is becoming a significant question. The present study aims to compare the assessment of work ability based on the use of the Work Ability Index (WAI), a 7-item questionnaire, with another one based on the use of WAI’s first item, which consists in the worker’s self-assessment of his/her current work ability level as opposed to his/her lifetime best, this single question being termed “Work Ability score” (WAS).

**Methods:**

Using a database created by an occupational health service, the study intends to answer the following questions: could the assessment of work ability be based on a single-item measure and which are the variables significantly associated with self-reported work ability among those systematically recorded by the occupational physician during health examinations? A logistic regression model was used in order to estimate the probability of observing “poor” or “moderate” WAI levels depending on age, gender, body mass index, smoking status, position held, firm size and diseases reported by the worker in a population of workers aged 40 to 65 and examined between January 2006 and June 2010 (n=12389).

**Results:**

The convergent validity between WAS and WAI was statistically significant (rs=0.63). In the multivariable model, age (p<0.001), reported diseases (OR=1.13, 95%CI [1.11-1.15]) and holding a position mostly characterized by physical activity (OR=1.67, 95%CI [1.49-1.87]) increased the probability of reporting moderate or poor work ability. A work position characterized by the predominance of mental activity (OR=0.71, 95%CI [0.61-0.84]) had a favourable impact on work ability. These relations were observed regardless of the work ability measurement tool used.

**Conclusion:**

The convergent validity and the similarity in results between WAI and WAS observed in a large population of employed workers should thus foster the use of WAS for systematic screening of work ability. Ageing, overweight, decline in health status, holding a mostly physical job and working in a large-sized firm increase the risk of presenting moderate or poor work ability.

## Background

Industrialised countries are experiencing significant demographic changes as people live longer and have healthier life and this is particularly true in Europe. Demographic projections by Eurostat (2011) indicate that the old-age dependency ratio - the ratio of those outside the labour force to those of working age (15–64 yr)- will double from 25.9% on average (in the 27 European countries) in 2010 to 50.2% by 2050.By then, two people of working age will be needed to support one pensioner. This rapidly ageing population is thus presenting challenges to the age structure of the workforce and to the sustainability of social protection schemes. As a result, national authorities in most European countries are trying to promote work ability throughout the working life, considering changes in the legal age for retirement, and preparing their citizens to a world in which everybody will stay longer on the labour market.

In this context, it is of utmost importance to identify simple ways to monitor work ability in the working population on a regular basis. The Finnish Institute of Occupational Health (FIOH) played a pioneering role during the 1980’s when it developed a generic tool to assess work ability, the so-called “Work Ability Index” (WAI); it considers the workers’ self-assessed work ability in relation to work requirements, health status and the worker resources [1]. WAI has since then been widely disseminated and is nowadays the most commonly used tool for measuring work ability [2].

It has been shown in several studies performed in various professional groups that age, obesity, lack of physical activity during spare time, low musculoskeletal ability, high mental requirements, lack of autonomy and heavy physical workload, all have a negative impact on the WAI level [2]. Other studies have demonstrated that WAI also has a predictive value, a low WAI level (or a level declining over time) increasing the probability of sick leaves [3-5], of early retirement [6-8] and even of worker decease [6].

Taking into consideration the WAI predictive validity and striving to improve the employment rate among workers aged over 55, the largest occupational health service in Luxembourg - called “Service de Santé au Travail Multisectoriel” (STM) - decided as from 2005 to use WAI within the framework of regular monitoring of workers’ health; this enabled the creation of a substantial database of WAI levels concerning its affiliated workers.

Based on the analysis of this database, the present study aims to answer the following questions:

1) Could the assessment of work ability be based on the use of a single item of WAI, the first one, which consists in self-assessment by workers of their current work ability level by comparison with the highest work ability experienced during their career, this question being termed “Work Ability score” (WAS) by the designers of the method [9]? Assessing work ability from this single question appears seducing on the grounds that the assessor has not to check the categorisation of the job function reported by the worker and it is also more understandable by the persons surveyed; some other WAI items, the 3^rd^ one - asking for the number of diagnosed diseases or the 7^th^ one – assessing psychological resources – are for instance often not well understood and may be left unanswered [10-15]. In addition, the 1^st^ item of WAI has high discriminating power (the highest) over the entire index [16]. This measure would in theory be easier both to implement and interpret in population surveys and could be carried out at a lower cost [13,17]. However, before possibly adopting this simplified procedure, one needs to assess its validity when compared to the full WAI. To date, such an analysis has only been performed in a relatively limited population of long-term disabled workers [13], in a sample of the general population in Finland [9] and in a group of Dutch construction workers [18].

2) Are age, body mass index, daily smoking, health status, firm size and type of work function determinants of the WAI level?

The answers to these two research questions would provide useful information when drawing guidelines for the national policy of promotion of active ageing which the Luxembourg government wishes to put in place.

## Methods

### Study design and setting

In the Grand-Duchy of Luxembourg, all firms must be affiliated with an occupational health service. The frequency of medical examinations is a function of the occupational hazards identified for each work position. STM performs yearly a medical follow-up of over 60,000 workers, which amounts to a third of the workers affiliated with the organization. Since 1997, STM has been using a system of computerized medical records (CMR) which allows structuring the collection of medical data and facilitates their archiving. In 2005, benefitting from the financial support of the European Social Fund, STM started using WAI during medical examinations. The medical assistant handed the workers an explanation letter and a WAI questionnaire and requested them to complete it in an unsupervised manner and then to hand it back to the occupational physician. These questionnaires were subsequently entered in the CMR by a specifically-trained nursing team.

The present study, of retrospective and multicentric character, concerns all workers employed by firms affiliated with STM, aged 40 to 65 (inclusive), who were examined by an occupational physician between January 1 2006 and June 30 2010 in the medical centres of Esch and Luxembourg and who agreed to fill in the questionnaire. For each worker, the WAI data collected were linked to other health data available in the CMR.

STM obtained approval of the National Commission for data protection (CNPD) for the creation of the medical database which included WAI data. The data extracted from this database for the purpose of this study were previously anonymized with an identification number which was different from the worker’s national social security number.

### Participants

Throughout the 54-month period of data-gathering, 31,959 workers aged 40 to 65 were examined in the 2 centres taking part in the study. From these, 17,900 (56.0%) handed a WAI questionnaire to the occupational physician. Afterwards, two choices were possible: use multiple imputations on the missing data or select only the fully-filled questionnaires. As multiple imputations also lead to measurement errors, the second option was selected. After the cleaning of the database, 12,839 full questionnaires were selected as the study sample database.

In order to detect a possible selection bias in the sample, a comparison between respondents and non-respondents was performed using the age, gender and BMI variables. The phi statistic which takes the “large sample size” effect into consideration was used [19] and the threshold of 0.50 was selected as recommended by the author for large samples.

### Variables

The study took into consideration WAI and WAS as dependent variables on the one hand, and as explanatory variables on the other hand a set of variables selected on the basis of literature review, their availability in the CMR and their estimated reliability during their collection by the physicians.

a. Assessment of work ability:

Work ability was measured by means of the Work Ability Index (WAI) which consists in a 7-part self-assessment: current ability, work ability in relation to physical and mental demands of the job, reported diagnosed diseases, estimated impairment due to health status, sick leave over the last 12 months, self-prognosis of work ability in the 2 years to come and mental resources of the individual. The WAI measured in this way ranges from 7 to 49 points and 4 categories have been suggested to describe WAI levels: poor [7-27], moderate [28-36], good (37-43) and excellent (44-49) [1].

The Work Ability score (WAS) consists in the worker’s self-assessment of his/her current ability compared to the lifetime best. It ranges from 0 to 10. The designers of the method [9] suggested the same type of categorization as for WAI, namely: poor (0–5 points), moderate [6,7], good [8,9], excellent [9].

b. Explanatory variables:

The selected explanatory variables were age, gender, weight and height as measured during the medical examination, tobacco consumption, diseases as recorded by the occupational physician in the worker’s medical file, workplace occupied (selected in a drop-down menu among 200 different choices), and the number of workers employed by the firm, the latter variable being subdivided into 4 classes corresponding to those used in the national labour legislation.

Regarding diseases, the CMR structure allows a distinction between minor diseases and those considered as major; only the latter have been taken into account in the analysis. Diseases were classified as major by a group of experts in occupational medicine and ergonomics, due to their potential impact on the current or future health of the worker and because they require systematic follow-up. The health status of each worker was estimated on the basis of the total number of recorded major diseases.

### Data-processing

The variables raw values were categorized into 2 or more classes for statistical purposes. Body mass index (BMI) was categorised in accordance with the recommendations of the World Health Organization (WHO) [20] (underweight <18.50, normal [18.50-24.99], overweight > 25.00, obesity > 30.00). Classification of smoking habits was based on Fagerström’s approach [21] (non-smoker, former smoker, 1 to 10 cigarettes, 11 to 20 cigarettes, 21 to 30 cigarettes, > 30 cigarettes). The major diseases were grouped according to system and the analysis was performed on the three disease categories which in practice raise the most difficulties in terms of job reintegration: functional and ischemic cardiac diseases, musculoskeletal disorders and mental diseases [9].

The 200 professional activities listed in the database were classified by 3 experts (2 occupational physicians and 1 ergonomist who had perfect knowledge of the Luxembourg typology in the area of naming work positions) into one of the 3 categories defined by the WAI mode of computation: predominantly physical functions, predominantly mental functions or mixed functions [22].

Quality control of the capture of WAI forms was performed: 500 worker forms were selected at random and re-entered in a secondary database. The concordance between the two capture stages (i.e. between the base analysed in the study and the secondary one) of the answers to each item was verified by means of Kendall’s tau-b test: depending on the item considered, the concordance ranged from 0.90 to 0.95.

### Statistical analysis

Descriptive statistics were applied to all the variables in the database.

#### Research question 1 - Comparison of WAI and WAS

The convergent validity of an instrument measures the degree of similarity between the ratings of that instrument and those of another instrument, supposed comparable [23]. To estimate the adequacy of substituting WAS to WAI in the assessment of work ability, the convergent validity between the two instruments was tested by evaluating the Spearman correlation between WAS and WAI levels; this analysis was carried out with data grouped into 4 categories (“poor”, “moderate”, “good” and “excellent”).

#### Research question 2 - Determinants of WAI and WAS

To analyse the determinants of WAI and of WAS, two logistic regressions were performed. A first regression was used to assess the probability of observing a WAI level lower or equal to 36 (“poor” and “moderate” classes), the reference category being a level above 36 (“excellent” and “good” classes). The same method was used to assess the probability of observing a WAS level lower than or equal to 7 (“poor” and “moderate” classes), the reference category being a level above 7 (“excellent” and “good” classes).

Independent variables were included in the logistic regression model according to their significance level in the univariable analysis (p<=0.20) and according to their lack of collinearity. Then, these variables were tested in the model using the backward selection method. The second-order interactions were tested (Wald test) and taken out of the model if they did not prove significant (p>0.10).To determine if the continuous variable had to be dichotomised, its linearity was also checked (Box-Tidwell Transformation Test). The final results were considered significant at the 5% significance level (P<0.05).

## Results

### Comparison respondents/non respondents

When comparing the characteristics of the 12,839 respondents to those of the 14,059 non-respondents, the two populations appeared different on first analysis: respondents were on average slightly younger, more likely to present normal weight (34.5% vs. 32.2%) and were more predominantly men (73.3% vs. 61.9%). Taking into account the large size of both populations, the analysis yielded phi values (size effects) inferior to 0.50 for the three variables studied (phi age = 0.05, phi gender = 0.12 and phi BMI = 0.03), demonstrating the lack of a significant association between these variables and the fact of being respondent [19]. This was reassuring as regards the possibility of a selection bias, although it did not, of course, rule it out.

### Descriptive analyses

Respondents’ WAI stood on average at 41.01 (SD = 6.23; median: 42) and WAS averaged 8.57 (SD = 1.57; median: 9), meaning in both cases a “good” work ability level according to the method.

Table 1 describes the distribution of individual and occupational variables within this population. Respondents (n=12,839) were predominantly men (73.3%) and were aged 47 on average (SD = 5.21). Overweight was detected in 40.6% of the workers and obesity in 24.0% of them. Nearly a quarter of them were active smokers and 9.9% smoked more than one pack of cigarettes a day. Musculoskeletal disorders were reported by 38.3% of the workers, mental disease by 10.3% and a cardiac pathology by 3.5%. The work positions occupied by the workers involved activities predominantly physical in 32.8% of the cases, predominantly mental in 21.9%, and what experts call “mixed” ones in 45.3%.

**Table 1:** Distribution of individual and professional variables in the sample (n= 12,839) (Figures rounded to one decimal)

**Variable**	**Categories**	**N**	**%**
WAI score	Excellent	5365	41.8
	Good	5027	39.2
	Moderate	1897	14.8
	Poor	550	4.3
Gender	Male	9412	73.3
Age	[40–45]	5471	42.6
	[45–50]	3856	30.0
	[50–55]	2308	18.0
	[55–60]	1008	7.9
	[60–65]	196	1.5
BMI	Underweight (< 18.5)	119	0.9
	Normal [18.5-25]	4425	34.5
	Overweight [25-30]	5214	40.6
	Obesity (≥ 30)	3076	24.0
	*Missing values*	5	
Smoking habits	Non-smoker	9222	71.8
	Former smoker	538	4.2
	from 1 to 10 cigarettes	753	5.9
	from 11 to 20 cigarettes	1053	8.2
	from 21 to 30 cigarettes	836	6.5
	Over 30 cigarettes	437	3.4
Type of position	Physical	4208	32.8
	Mental	2809	21.9
	Mixed	5822	45.3
FICD	Presence	445	3.5
MSD	Presence	4915	38.3
Mental diseases	Presence	1317	10.3
Size of firm staff	1-9	1972	17.1
	10-49	3871	33.6
	50-249	3887	33.7
	Over 250	1798	15.6
	*Missing values*	*1311*	

BMI in kg/m^2^, FICD: Functional and ischemic cardiac diseases, MSD: Musculoskeletal disorders.

#### Research question 1 - Comparison of WAI and WAS

The Spearman correlation between WAS and WAI levels was statistically significant (rs=0.63; p < 0.001); this level of correlation indicates more than acceptable convergent validity between the two instruments [23].

#### Research question 2 - Determinants of WAI and WAS

Table 2 shows the univariable and multivariable analyses of the probability of presenting a so-called “moderate” or “poor” WAI based on the individual and occupational factors studied. In the univariable analysis, this probability was lower in male workers (OR=0.88; 95%CI [0.80-0.97]) and higher in former smokers, in smokers of 21–30 cigarettes (OR=1.29; 95%CI [1.09-1.54]) and smokers of 31 cigarettes or more (OR=1.67; 95%CI [1.35-2.08]) compared to non-smokers; it was also higher in overweight (OR=1.15; 95%CI [1.03-1.27]) and obese (OR=1.39; 95%CI [1.24-1.56]) workers. After adjustment for the other variables, ‘gender’ and ‘daily smoking’ were removed from the model. The table furthermore indicates that the association between “moderate” or “poor” WAI and weight status was no longer significant in multivariable analysis (p=0.08).

**Table 2:** Odds ratio (OR) and 95% confidence intervals (CI) for individuals and professional factors associated with Work Ability Index – WAI (moderate-poor) vs (excellent-good)

**Variable**	**N (%)**	**Univariable analysis**			**Multivariable analysis**		
		**OR**	**95% CI**	**P-value**	**OR**	**95% CI**	**P-value**
Gender*				**0.01**			
Female	702 (20.5)	1					
Male	1745 (18.5)	**0.88**	**[0.80-0.97]**				
**Age**				**<0.001**			**<0.001**
[40–45]	760 (13.9)	1			1		
[45–50]	687 (17.8)	**1.34**	**[1.20-1.50]**		**1.27**	**[1.11-1.15]**	
[50–55]	637 (27.6)	**2.36**	**[2.10-2.66]**		**2.08**	**[1.81-2.40]**	
[55–60]	320 (31.7)	**2.88**	**[2.47-3.36]**		**2.82**	**[2.35-3.38]**	
[60–65]	43 (21.9)	**1.74**	**[1.23-2.46]**		**2.68**	**[1.81-3.96]**	
**BMI**				**<0.001**			0.08
Normal	752 (17.0)	1			1		
Underweight	23 (19.3)	1.17	[0.74-1.86]		1.02	[0.55-1.86]	
Overweight	991 (19.0)	**1.15**	**[1.03-1.27]**		1.06	[0.94-1.20]	
Obesity	681 (22.1)	**1.39**	**[1.24-1.56]**		**1.20**	**[1.04-1.38]**	
**Daily smoking habits**				**<0.001**			
Non-smoker	1670 (18.1)	1					
Former smoker	133 (24.7)	**1.49**	**[1.21-1.82]**				
From 1 to 10 cigarettes	131 (17.4)	0.95	[0.78-1.16]				
From 11 to 20 cigarettes	209 (19.8)	1.12	[0.95-1.32]				
From 21 to 30 cigarettes	186 (22.2)	**1.29**	**[1.09-1.54]**				
> 30 cigarettes	118 (27.0)	**1.67**	**[1.34-2.08]**				
**Health status**							
Number of diseases		**1.27**	**[1.25-1.29]**	**<0.001**	**1.13**	**[1.11-1.15]**	**<0.001**
FICD / Yes	185 (41.6)	**3.19**	**[2.63-3.87]**	**<0.001**	**1.43**	**[1.11-1.84]**	**0.01**
MSD / Yes	1603 (32.6)	**4.06**	**[3.70-4.46]**	**<0.001**	**2.39**	**[2.12-2.70]**	**<0.001**
Mental diseases / Yes	612 (46.5)	**4.58**	**[4.07-5.16]**	**<0.001**	**2.54**	**[2.18-2.96]**	**<0.001**
**Type of work function**				**<0.001**			**<0.001**
Mixed	1036 (17.8)	1			**1**		
Physical	1121 (26.6)	**1.68**	**[1.52-1.85]**		**1.67**	**[1.49-1.87]**	
Mental	290 (10.3)	**0.53**	**[0.46-0.61]**		**0.71**	**[0.61-0.84]**	
**Firm size**				**<0.001**			**0.03**
1-9	290 (14.7)	**0.56**	**[0.47-0.66]**		**0.76**	**[0.63-0.91]**	
10-49	672 (17.4)	**0.68**	**[0.59-0.78]**		**0.84**	**[0.72-0.98]**	
50-249	722 (18.6)	**0.74**	**[0.64-0.84]**		0.87	[0.74-1.01]	
>=250	426 (23.7)	1			1		

* Taken into account in the multivariable analysis but not part of the equation following Wald’s step-by-step descending method (the variable effect is not high enough to be kept in the model).

To help to read the table, let us comment on one example: in the multivariable analysis, taking the age category [40–45] as a reference, the probability to obtain a poor to moderate WAI is significantly higher in the other age categories.

Several other factors however had a negative influence on the WAI index both in univariable and multivariable analyses: age (p<=0.001), presence of major diseases, be they cardiovascular (OR=1.43; 95%CI [1.11-1.84]), musculoskeletal (OR=2.39; 95%CI [2.12-2.70]) or mental (OR=2.54; 95%CI [2.18-2.96]) and holding a predominantly physical work function (OR=1.67; 95%CI [1.49-1.87]). On the contrary, holding a mostly mental function had a favourable impact (OR=0.71; 95%CI [0.61-0.84]) and so did working in a firm employing fewer than 10 workers (OR=0.76; 95%CI [0.63-0.91]) or between 10 and 49 workers (OR=0.84; 95%CI [0.72-0.98]) in comparison to those working in large firms (>=250 staff).

Table 3 shows univariable and multivariable analyses of the probability of presenting a so-called “moderate” or “poor” WAS level based on individual and occupational factors. The univariable analysis highlighted the same associations as those observed for the WAI index with the exception of the association between work ability and gender which in this case was totally absent.

**Table 3:** Odds ratio (OR) and 95% confidence intervals (CI 95) for individual and professional factors associated with Work Ability score – WAS (moderate-poor) vs (excellent-good)

**Variable**	**N (%)**	**Univariable analysis**			**Multivariable analysis**		
		**OR**	**95% CI**	**P-value**	**OR**	**95% CI**	**P-value**
**Gender**				0.99			
Female	620 (18.1)	1					
Male	1702 (18.1)	1	0.90 -1.11				
**Age**				**< 0.0001**			**< 0.0001**
[40–45]	733 (13.4)	1			**1**		
[45–50]	656 (17.0)	**1.33**	**1.18-1.49**		**1.25**	**1.10-1.42**	
[50–55]	576 (25.0)	**2.15**	**1.90-2.43**		**1.87**	**1.63-2.15**	
[55–60]	309 (30.6)	**2.86**	**2.45-3.34**		**2.73**	**2.29-3.26**	
[60–65]	48 (24.5)	**2.10**	**1.50-2.93**		**3.09**	**2.15-4.44**	
BMI*				**< 0.0001**			
Normal	721 (16.3)	1					
Underweight	17 (14.3)	0.86	0.51-1.44				
Overweight	972 (18.6)	**1.18**	**1.06-1.31**				
Obesity	612 (19.9)	**1.28**	**1.13-1.44**				
**Daily smoking habits**				**< 0.0001**		0,06	
Non-smoker	1611 (17.5)	1			1		
Former smoker	110 (20.5)	1.21	0.98-1.51		1.27	0.97-1.66	
From 1 to 10 cigarettes	112 (14.9)	0.83	0.67-1.02		1.30	0.92-1.85	
From 11 to 20 cigarettes	219 (20.8)	**1.24**	**1.06-1.45**		0.99	0.70-1.40	
From 21 to 30 cigarettes	167 (20.0)	1.18	0.99-1.41		**1.47**	**1.08-2.00**	
> 30 cigarettes	103 (23.6)	**1.46**	**1.16-1.83**		1.19	0.86-1.65	
**Health status**							
Number of diseases		**1.18**	**1.16-1.19**	**< 0.0001**	**1.09**	**1.07-1.10**	**< 0.0001**
FICD / Yes	163 (36.6)	**2.74**	**2.25-3.34**	**< 0.0001**	**1.45**	**1.14-1.85**	**0.003**
MSD / Yes	1380 (28.1)	**2.89**	**2.64-3.17**	**< 0.0001**	**1.95**	**1.74-2.20**	**< 0.0001**
Mental diseases / Yes	501 (38.0)	**3.27**	**2.90-3.70**	**< 0.0001**	**1.98**	**1.70-2.30**	**< 0.0001**
**Type of work function**				**< 0.0001**			**< 0.0001**
Mixed	960 (16.5)	1			**1**		
Physical	1043 (24.8)	**1.67**	**1.51-1.84**		**1.65**	**1.48-1.85**	
Mental	319 (11.4)	**0.65**	**0.57-0.74**		**0.78**	**0.67-0.91**	
Firm size*				**< 0.0001**			
1-9	307 (15.6)	**0.70**	**0.59-0.82**				
10-49	623 (16.1)	**0.72**	**0.63-0.83**				
50-249	681 (17.2)	**0.80**	**0.70-0.92**				
>=250	377 (21.0)	1					

* Taken into account in the multivariable analysis but not part of the equation following Wald’s step-by-step descending method.

To help to read the table, let us comment on one example: in the multivariable analysis, taking the age category [40–45] as a reference, the probability to obtain a poor to moderate WAS is significantly higher in the other age categories.

The negative associations noticed in multivariable analysis were similar to those described for WAI concerning age (p<=0.001), number of major diseases reported (p<=0.001), presence of cardiovascular diseases (p<=0.003), musculoskeletal disorders (p<=0.001) or mental diseases (p<=0.001), as well as holding a predominantly physical work function (p<=0.001). There were however two differences when compared to the associations described in Table 2: firm size was removed from the model, and the 21–30 cigarettes’ category had a negative influence on WAS score (OR=1.47; 95%CI [1.08-2.00]).

## Discussion

The present study goal was to compare the assessment of work ability based on the use of the Work Ability Index (WAI) to the one based on the use of the first item of WAI, this single question being termed “Work Ability score” (WAS), in a population of workers occupying a wide variety of jobs or functions. The non-participation rate in WAI assessment was relatively high (44%) among the workers concerned but does not seem to have induced any recruitment bias insofar as the demographic characteristics of the respondents overlap with those of the non-respondents. This high non-participation rate could most probably be ascribed to difficulties in administrative management experienced within the collaborating medical centres.

### Research question 1 - Validity of WAS compared to WAI and usefulness of WAS

The relative merits of using either a single-item measure or a multiple-item (or scale) measure have been discussed at great length in the occupational psychology literature for assessing job satisfaction [24,25]. Although job satisfaction and work ability are constructs of a different nature, they are both complex constructs with multifaceted determinants. It is thus tempting to hypothesize, as these authors did for job satisfaction, that simply combining 7 pre-selected items or dimensions of work ability to obtain an overall index of work ability may in some cases exclude other significant aspects of the man–machine interaction that may be very influential in determining the worker’s own perception of his/her work ability. One could however argue that for assessing work ability, taking into account the number of diseases the worker is currently encountering (WAI item 3) and the importance of sick leave in the last 12 months (WAI item 5) should substantially increase the content validity of WAI in comparison to the WAS single-item measure.

In the present study originating in occupational health practice, the comparison between WAI and WAS was first guided by cost-effectiveness considerations. As stated by Wanous et al. for job satisfaction measures [24], a single-item measure is shorter in length, requires less time to complete and is more likely to be completed by the employee. Since the introduction of WAI in the medical surveillance routine, STM has experienced difficulties in the use of this tool, whether for the occupational health service (need for external expertise when defining work function categories and for a high degree of rigor when doing the data capture and calculating the index) or for the workers themselves. When the worker does not have a good understanding of the WAI aim, he/she can only with difficulty answer correctly all questions pertaining to the 7 items, which could account for the high proportion of questionnaires not completely filled in (28.3%) in the present study. Missing values were particularly frequent (> 15%) for WAI item 4 (estimation of work impairment due to the diseases), item 5 (sickness absence), item 6 (prognosis of ability over 2 years) and item 7 (psychological resources).

The results obtained in the study seem to indicate that using the single-item approach instead would not deteriorate the validity of the work ability information collected. The level of convergent validity observed between WAS and WAI was quite satisfactory (rs = 0.63) and of the same order of magnitude as the correlation obtained for job satisfaction measures [24]. In addition, the present analysis shows that the assessment based on WAS (1 item) highlighted the same factors of increase or reduction in work ability as did the 7-item WAI, with the exception of the effect of firm size (not observed with WAS in the multivariable model). WAS therefore appears as a tool to be used in priority in the future as its user-friendliness brings in a clear advantage for a systematic application during medical examinations performed within the field of occupational health care.

### Research question 2 – Determinants of work ability

#### Relation with individual factors

The relations observed between work ability and the individual variables generally corroborate those reported in the literature. The results show a strong association between ageing and the decline in work ability, whether be it assessed by WAI or by WAS; numerous studies have indeed demonstrated that young workers estimate their work ability at a higher level than older ones [26-31]. In the results obtained, the relation observed is nevertheless not fully linear as work ability assessed in 60-65-year olds was better than in 55-59-year olds (Tables 2 and 3). The limited size of the older worker group when compared to the other age groups supports the hypothesis of a “healthy worker” effect, healthier individuals being able to stay longer on the labour market, a well-described phenomenon in several industries [3].

The data analysed did not highlight any relation between gender and work ability, an observation in line with the systematic review done by van den Berg et al. (2008) [2]. It is worth mentioning however that this relation varied according to the measurement tool used, either WAI or WAS. On the basis of WAI measurement, the probability of low or poor work ability was higher in women (significantly in univariable and not reported in multivariable). Yet, this association disappeared when ability was measured using WAS. Such a discrepancy between the two methods has also been reported in the Finnish health survey [9]. The authors suggested that the decrease in WAI but not in WAS level in women could be accounted for by a higher number of sick leaves and days of absence but also by lower psychological resources [9].

The population studied in Luxembourg included a high proportion of overweight workers and, in line with the observations reported in other studies [2,26,32-34], the present results suggest that those workers are more at risk of presenting moderate or poor WAI and that this risk increases as a function of the excess weight; but it must be noted that this association was either not significant (p=0.08) or not included in the multivariable model. This could be due to the inclusion in the model of the ‘number of diseases’ variable, the link between overweight status and several pathologies, especially cardiovascular ones, being well established. Another unhealthy behaviour significantly increased the risk of presenting low or poor work ability: a dose-effect relationship was observed in univariable analysis with the number of smoked cigarettes. This association was however not significant in the multivariable model, and this could possibly be ascribed to the importance of the diseases variable in the model. In the literature, an association between work ability and workers’ smoking habits was reported as significant in a single study only [26].

In the present study, the relation between health status and work ability has been explored on the basis of the number of major diseases recorded by the occupational physician. Mental diseases and musculoskeletal disorders exerted the strongest negative influence on WAI; the association with cardiovascular diseases was not so clear unlike the observations made in the Finnish health survey [9].

The prevalence of musculoskeletal disorders in the studied population (38.3%) seems in line with the results of the European survey in which 24.7% complained about backache and 22.8% about muscle pain [35]. Regarding mental health problems (psychosis, anxiety, addictive behaviour,…), the rate observed in the sample (10.3%) appears relatively low when compared to the rates reported in the European survey for stress (22.3%), irritability (10.5%), or anxiety (7.8%). Nevertheless, given the possible impact of such self-reported mental health problems on the “fit for work” decision to be issued by the occupational physician, some under-reporting bias could be hypothesized.

### Relation with occupational factors

This study highlighted significant differences in self-estimated work ability according to the type of work function held. Workers assigned to a predominantly mental function presented higher work ability levels than those assigned to a mostly physical function. This trend was observed both for WAI and WAS levels, and this is in line with literature data [9,27,36,37]. This association could not only reflect the detrimental effects of chronic exposure to biomechanical and postural stress in physical jobs but also the impact of low work control and poor job content [27].

Firm size, or in other words the number of workers employed, also influenced work ability as estimated by WAI. The probability of presenting a WAI level defined as moderate to poor was indeed lower in firms employing less than 50 workers. This observation could be linked to a more favourable relational environment in small and very small (<10 workers) firms. Literature data however does not provide any information on this issue.

#### Strengths and limitations of the study

A strength of the study lies not so much in the size of the population sample studied (over half of the workers in Luxembourg) but in the wide variety of professional sectors represented. Not all sectors were represented however: the STM service is not empowered to monitor occupational health in such sectors as banks and insurance companies or even hospitals. These sectors have their own occupational health service.

From a methodological point of view, the large sample of WAI data available made the exclusive selection of fully complete questionnaires a better option than the use of substitution algorithms for missing values.

Another strength of the study lies in its inscription in the real practice conditions of workers’ health surveillance as performed by an occupational health service; the study showed the difficulty of applying a standardized and systematic process for work ability assessment with more than 25 different nurses and physicians being involved.

The study design also has some limitations: the completion of the questionnaire being made on a voluntary basis, without direct supervision, one cannot rule out the idea that less educated workers decided not to fill in the questionnaire or when filling it in, failed to provide information for all the items. Another potential limitation has to do with the quality of medical variables. The CMR used in the STM service was not primarily intended for epidemiological studies and the lack of standardized anamnesis implies that the data-capture and exploitation of the CMR might have been influenced by features specific to each medical examiner.

Another limitation arises from the asymmetric gender distribution in the studied sample: any extrapolation of the observations of this study to other populations of workers with balanced gender distribution would require utmost caution.

## Conclusion

This study shows that work ability, be it measured by WAI or by WAS in a large population of employed workers in Luxembourg, is associated with the same independent variables as those pointed out in other worker populations [2]. Ageing, overweight, decline in health status and holding a mostly physical professional function increase the risk of presenting moderate or poor work ability.

The convergent validity and the similarity in results between these two tools should thus foster the use of the single item of WAS, the self-assessed current ability level in comparison to lifetime best, for a systematic screening of work ability in worker populations either within occupational health care or in public health surveys. Taking into account this study results, STM has decided to integrate WAS into the systematic anamnesis of all workers at each medical examination; any decrease in subjective work ability will thus be detected prospectively and will trigger a reanalysis of the working conditions and the implementation of a coaching approach for the worker concerned. With regard to primary prevention, STM will propose their affiliated enterprises specific prevention programmes which will focus on the two health problems most strongly linked to a low ability level: mental health and musculoskeletal disorders [38,39].

Some observations made in the present study would nevertheless deserve further studies in the future. In view of the rate of non-respondents noted (44%), the factors which can influence the participation in this type of assessment should be investigated. More particularly, one would need to assess the possible interaction between perceived health and participation: workers perceiving their health as declining could decide not to fill in the WAI questionnaire for fear of influencing the occupational physician’s decision concerning their fitness for work.

## Abbreviations

CMR: Computerized medical records; CNPD: National Commission for data protection; FICD: Functional and ischemic cardiac diseases; F.N.R.: National Research Fund; MSD: Musculoskeletal disorders; OR: Odds ratio; STM: Service de Santé au Travail Multisectoriel; WAI: Work Ability Index; WAS: Work Ability score; WHO: World Health Organization.

## Competing interests

The authors declare that they have no competing interests.

## Authors’ information

EFM: Researcher, Public Health Department, Liège University (Belgium) and Centre for Health Studies, CRP-Santé, Luxembourg

BV: Biostatistician, Competences Centre for Methodology and Statistics, CRP-Santé, Luxembourg

MN: Medical director of the “Service de Santé au Travail Multisectoriel” (STM) (GD du Luxembourg)

LM-L: Director of the Centre for Health Studies, CRP-Santé, Luxembourg

CS: Associate Head of Unit, “Epidemiology and Public Health”, CRP-Santé, Luxembourg

MP: Director of the Occupational Health and Health Education unit (STES), Public Health Department, Liège University (Belgium)

## Authors’ contributions

MEF: main investigator, in charge of database quality assessment, categorisation of variables, statistical analysis (in close link with B.V.), writing of the first draft of the manuscript and its successive revisions. VB: data statistical analysis, revision of the Methods section. NM: initiating the study and defining its aims, supervising the day-to-day management of the computerized files within the STM Occup. Health Service, supervising the data extraction, directing the expert team for the classification of each of the 200 work functions, revision of the manuscript. M-LL: defining the study aims, revision of the manuscript. SC: methodological advice, revision of the manuscript. PM: defining the study aims (with MN and LML), supervising the main investigator, first revision of each successive draft of the manuscript. All authors read and approved the final manuscript.

## Pre-publication history

The pre-publication history for this paper can be accessed here:

http://www.biomedcentral.com/1471-2458/13/305/prepub
